# Dendrowardol C, a novel sesquiterpenoid from *Dendrobium wardianum* Warner

**DOI:** 10.1007/s13659-013-0024-9

**Published:** 2013-04-24

**Authors:** Wei-Wei Fan, Feng-Qing Xu, Fa-Wu Dong, Xiao-Nian Li, Yan Li, Yu-Qing Liu, Jun Zhou, Jiang-Miao Hu

**Affiliations:** 124 State Key Laboratory of Phytochemistry and Plant Resources in West China, Kunming Institute of Botany, Chinese Academy of Sciences, Kunming, 650201 China; 224 University of Chinese Academy of Sciences, Beijing, 100049 China

**Keywords:** *Dendrobium wardianum* Warner, sesquiterpenoid, dendrowardol, X-ray

## Abstract

Dendrowardol C (**1**)—a novel sesquiterpenoid, with an unprecedented 4/5/6/6 tetracyclic carbon backbone, together with two known cyclopacamphane-type sesquiterpenoids; dendronobilin I (**2**) and dendrobane A (**3**) were isolated from the stems of *Dendrobium wardianum* Warner. The structure of **1** was established on the basis of spectroscopic data and the absolute configuration was determined by single-crystal X-ray diffraction crystallography. The hypothetical biosynthetic pathway of **1** was postulated. Compound **1** showed no cytotoxic activity against human tumor cell lines HL-60, SMMC-7721, A-549, MCF-7, and SW480.

**Electronic Supplementary Material:**

Supplementary material is available for this article at 10.1007/s13659-013-0024-9 and is accessible for authorized users.

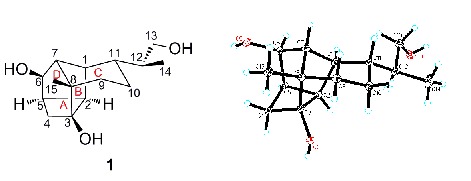

## Introduction

The stems of several *Dendrobium* species (Orchidaceae) are used in traditional Chinese medicine mainly for nourishing the stomach, promoting secretion of saliva, and reducing fever.[1] *Dendrobium wardianum* Warner is an endemic plant distributed mainly in southern Yunnan Province, China and some southeast Asian countries, i.e. Myanmar, Bangladesh, and Thailand.[2] This plant was rarely used as "Shi-Hu" ever and previous chemical investigation on this plant has led to the isolation of an picrotoxane-type alkaloid, dendrowardine.[3] Recently, we have isolated three sesquiterpenoids from the stems of *D. wardianum* Warner[4] and continual chemical investigation of the same collection of this plant led to the isolation of one novel sesquiterpenoid, dendrowardol C (**1**) with an unprecedented 4/5/6/6 tetracyclic ring system, together with two known cyclopacamphane-type sesquiterpenoids. The new structure was determined on the basis of extensive spectroscopic analysis and the X-ray crystallographic diffraction analysis, while the known sesquiterpenoids were identified as dendronobilin I (**2**)[5] and dendrobane A (**3**)[6] by comparison with the literatures. In addition, the hypothetical biosynthetic pathway of **1** was postulated.

## Results and Discussion

Compound **1** was isolated as a colorless crystal (MeOH). Its molecular formula ($C_{15}H_{24}O_3$), was established on the basis of

**1** **2** **3**

HRESIMS from the $[M + Na]^+$ ion at *m/z* 275.1627 (calcd. for 275.1623), indicating four degrees of unsaturation. The $^1H$ NMR spectrum of **1** (Table 1) indicated the existence of two methyl groups [$\delta_H$ 1.71 (3H, s), 1.19 (3H, d, $J = 6.6$ Hz)]. The $^{13}C$ NMR (DEPT) spectra of **1** (Table 1) revealed 15 carbon signals arising from two quaternary carbons (one oxygenated), seven methines (one oxygenated), four methylenes (one oxygenated) and two methyls. Since no C=O and C=C double bonds were dectected according to $^{13}C$ NMR and IR data, a tetracyclic structure was required for **1** to fulfill the four degrees of unsaturation. The comparison of $^1H$ and $^{13}C$ NMR spectra data of **1** (Table 1) with those of dendronobilin I (**2**) suggested that **1** and **2** were similar in rings B and C, indicating the similar structure of **1** and **2**. For the cyclopacamphane-type sesquiterpene in *Dendrobium* species, C-3 is usually a quaternary carbon without oxygen and C-15 is constantly a methyl or an oxygenated methylene.[5–7] Interestingly, an oxygenated quaternary carbon ($\delta_C$ 78.1) and a methylene carbon ($\delta_C$ 35.2) signals appeared in $^{13}C$ NMR (DEPT) spectrum of compound **1**. From the HSQC spectral data of **1**, twenty-one protons were assigned unambiguously to thirteen carbons, respectively. The partial structure (rings B and C)

*To whom correspondence should be addressed. E-mail: hujiangmiao@mail.kib.ac.cn

**Table 1.** $^1$**H NMR data of compound 1 and** $^{13}$**C NMR data of compounds 1 and 2 in pyridine-*d*$_5$**

| No. | **1**[a] $\delta_H$ | **1**[a] $\delta_C$ | **2**[b] $\delta_C$ |
|---|---|---|---|
| 1 | 2.12 (br. s) | 44.6 d | 41.9 d |
| 2 | 2.89 (br. d, 4.2) | 53.9 d | 19.3 d |
| 3 | | 78.1 s | 30.8 s |
| 4a | 2.25 (br. t, 8.8) | 35.2 t | 23.9 d |
| 4b | 3.08 (br. d, 9.7) | | |
| 5 | 2.41 (m) | 31.3 d | 78.1 d |
| 6 | 4.40 (m) | 76.2 d | 52.4 d |
| 7 | 1.91 (br. s) | 55.6 d | 44.9 s |
| 8 | | 46.3 s | 35.6 t |
| 9a | 1.40 (m) | 38.4 t | |
| 9b | 2.18 (overlapped) | | 23.7 t |
| 10a | 1.72 (overlapped) | 25.1 t | 38.6 d |
| 10b | 2.16 (overlapped) | | |
| 11 | 1.89 (m) | 38.5 d | 39.9 d |
| 12 | 1.75 (overlapped) | 40.7 d | 65.7 t |
| 13a | 3.77 (dd, 10.4, 6.6) | 65.8 t | 15.5 q |
| 13b | 3.96 (dd, 10.4, 4.0) | | |
| 14 | 1.19 (d, 6.6) | 15.7 q | 22.7 q |
| 15 | 1.71 (s) | 19.8 q | 59.5 t |

[a] $^1$H NMR recorded at 500 MHz, $^{13}$C NMR recorded at 125 MHz; [b] $^{13}$C NMR recorded at 100 MHz.

was constructed by $^1$H-$^1$H COSY correlations of H-1/H-2/H-5/H-6/H-7, H-1/H-7, and H-9/H-10/H-11/H-1, and HMBC correlations from H-9 to C-7 and C-8, from Me-15 to C-7 and C-8. The HMBC (Figure 1) correlations of H-1, H-2 and H-4 ($\delta_H$ 2.25, 3.08) with C-3 ($\delta_C$ 78.1), as well as $^1$H-$^1$H COSY correlation of H-4/H-5, indicated the linkage of C-2/C-3/C-4, which established a four-membered ring C system. Furthermore, the cross-peak of Me-15/C-3 displayed the connecting of C-8/C-3. These data were in good agreement with the spectral data observed for C-3 (an oxygenated quaternary carbon) and C-4 (an methylene carbon). The other correlations in $^1$H-$^1$H COSY spectrum revealed the fragments of C-13/C-12/C-14 and C-11/C-12. Thus, the planar structure of **1** with a novel 4/5/6/6 tetracyclic ring system was proposed as shown in Figure 1.

**Figure 1.** Key $^1$H-$^1$H COSY, HMBC and ROESY correlations of **1**

The correlations of H-1/H-7, H-7/H-11, and H-1/H-6, were observed from the ROESY spectrum (Figure 1), which revealed that H-1, H-7, H-11 and OH-6 were $\alpha$-, $\alpha$-, $\alpha$-, $\beta$-oriented, respectively. Since C-3 (in ring A system) was established to be connected to C-8 (in ring C system) in the structure of **1** with a 4/5/6/6 tetracyclic carbon skeleton, Me-15, H-2, and H-5 should be on the same side as H-1 and H-6, possessing $\alpha$-orientation. Both Me-15 and H-5 were $\alpha$-oriented, which was also supported by the correlations of H-7/Me-15 and H-5/H-6 in the ROESY spectra. Thus, the relative configurations of all chiral carbons in the molecule except for C-12 were determined as the configuration of the stereocenter at C-12 could not be determined due to free rotation of this center with the C-11–C-12 bond[5].

To characterize the absolute configuration of **1**, a proper crystal was obtained using an applied single crystal X-ray diffraction with Cu K$\alpha$ radiation. As shown in Figure 2, the absolute configurations at C-1, 2, 3, 5, 6, 7, 8, 11, and 12 were deduced as 1*R*, 2*R*, 3*R*, 5*R*, 6*R*, 7*R*, 8*S*, 11*R*, and 12*S* based on X-ray crystallographic data (CCDC-896832).

**Figure 2.** X-ray structure of **1**

The presence of the two structurally related sesquiterpenoids (**1** and **2**) in the same plant implied that dendrowardol C (**1**) might be derived from the sesquiterpene cyclosativene.[8] The compound was oxidized to intermediate **a**, then dehydration under acidic conditions to intermediate **c**. After a Wagner-Meerwein rearrangement via C3–C4 bond-shift to give an intermediate,[9] cyclobutyl cation (**d**), which was then attacked by $H_2O$ as the nucleopilic reagent and then underwent deprotonation and get the compound **1** (as shown in Scheme 1).

Compound **1** was assayed for its cytotoxicity against five human cancer cell lines (HL-60, SMMC-7721, A-549, MCF-7, and SW480) by the MTT assay method, with DDP and taxol as positive controls. The results showed that compound **1** exhibited no significant cytotoxic activity against the above cell lines at the concentration of 40 $\mu$M.

**Scheme 1.** Plausible biogenetic pathway of **1**

## Experimental Section

**General Experimental Procedures.** Melting points were obtained on an X-4 micro melting point apparatus. Optical rotations were measured with a Horiba SEPA-300 polarimeter. UV spectra were obtained using a Shimadzu UV-2401A spectrometer. IR spectra were recorded on a Bruker FT-IR Tensor 27 spectrometer using KBr pellets. 1D and 2D NMR

spectra were recorded on Bruker Avance III 600 MHz, Bruker DRX-500 MHz, or AV-400 MHz spectrometers with TMS as the internal standard. Chemical shifts ($\delta$) are expressed in ppm relative to TMS. HREIMS was recorded on a Waters Auto Premier P776 spectrometer. Positive HRESIMS experiments were carried out on a VG Autospec-3000 spectrometer under 70 eV and a LCMS-IT-TOF (Shimadzu, Kyoto, Japan). Column chromatography (CC) was carried out using silica gel (200–300 mesh) and TLC was carried out on plates precoated with silica gel (10–40 $\mu$m, Qingdao Marine Chemical Ltd., Qingdao, China). RP-18 gel (40–63 $\mu$m, Merck, Darmstadt, Germany), and Sephadex LH-20 was purchased from Amersham Biosciences.

**Plant Material.** The *D. wardianum* Warner plants were purchased in August 2009 from Puer, Yunnan Province and identified by Dr. Xiao-Hua Jin of Institute of Botany, Chinese Academy of Sciences. A voucher specimen (No. Zsh-7) was preserved at the State Key Laboratory of Phytochemistry and Plant Resource in West China, Kunming Institute of Botany, CAS, China.

**Extraction and Isolation.** The fresh stems of *D. wardianum* Warner (50 Kg) were extracted with 95% EtOH (24 L × 3) and the ethanol solution was concentrated to a water suspension under reduced pressure by rotary evaporator and then partitioned between $CHCl_3$ and $HCl/H_2O$ (pH 2). The aqueous layer was adjusted to pH = 10 with 1 M sodium hydroxide solution and then extracted with $CHCl_3$ to give an alkaloidal extract (6.0 g). The aqueous phase was then subjected to macroporous resin (D101) chromatography to afford the crude water-soluble material (92.0 g). The water soluble material was subjected to silica gel column chromatography (CC) ($CHCl_3$/MeOH, 30:1, 20:1, 15:1, 10:1, 5:1, 0:1) to afford fractions I−VII. Fraction III (8.5 g) was subjected to CC over silica gel ($CHCl_3$-MeOH, from 20:1 to 2:1), Sephadex LH-20 chromatography (MeOH), RP-18 CC (MeOH/$H_2O$, 1:4−1:0) and further purified through recrystallization from MeOH to yield **1** (5 mg) and dendronobilin I (**2**) (4 mg). Similarly, fraction VI (8.9 g) was purified through repeated chromatography to afford dendrobane A (**3**) (2 mg).

**Dendrowardol C (1):** mp 193–194 °C; $[\alpha]_D^{20.7}$ + 48.64 (*c* 0.26, MeOH); UV (MeOH) $\lambda_{max}$ (log $\varepsilon$): 203 (2.59) nm; IR (KBr) $\nu_{max}$ 3499, 2960, 2935, 2900, 2871, 2828, 1456, 1381, 1283, 1269, 1032, 762 $cm^{-1}$; $^1H$ and $^{13}C$ NMR data, see Table 1; ESIMS *m/z* 275 $[M + Na]^+$, HRESIMS *m/z* 275.1627 (calcd. for $C_{15}H_{24}O_3Na$, 275.1623).

**Crystallographic Data of Dendrowardol C (1):** $C_{15}H_{24}O_3$, $M$ = 252.34; orthorhombic system, space group P212121, $a$ = 6.39120(1) Å, $b$ = 11.8518(2) Å, $c$ = 18.2414(3) Å, $\alpha = \beta = \gamma$ = 90°, $V$ = 1381.74(4) Å$^3$, $Z$ = 4, $d$ = 1.213 g/cm$^3$. A crystal of dimensions 0.90 × 0.19 × 0.18 mm$^3$ was used for measurement on a Bruker APEX DUO with a graphite monochromater, Cu Kα radiation. Reflections collected: 13935. Independent reflections: 13935/2522 [$R_{int}$ = 0.0384]. Completeness to θ = 69.59°:98.3%. The structure was solved by direct methods and refined by a full-matrix least squares on $F^2$. Final $R$ indices [$I > 2\sigma(I)$]: $R_1$= 0.0310, $wR_2$ = 0.0814. Flack parameter: 0.00(18). Crystallographic data for dendrowardol C (**1**) has been deposited at the Cambridge Crystallographic Data Centre as deposition number CCDC 896832. Copies of the data can be obtained free of charge on application to CCDC, 12 Union Road, Cambridge CB2 1EZ, UK (Tel: + 44 (0)1223 762911, e-mail: deposit@ccdc.cam.ac.uk).

**Cytotoxicity Assay.** The following human tumor cell lines were used: HL-60, SMMC-7721, A-549, MCF-7, and SW480. All the cells were cultured in RMPI-1640 or DMEM medium (Hyclone, Logan, UT), supplemented with 10% fetal bovine serum (Hyclone) at 37 °C in a humidified atmosphere with 5% $CO_2$. Cell viability was assessed by conducting colorimetric measurements of the amount of insoluble formazan formed in living cells based on the reduction of 3-(4,5-dimethylthiazol-2-yl)-2,5-diphenyltetrazolium bromide (MTT) (Sigma, St. Louis, MO). Briefly, 100 $\mu$L of adherent cells were seeded into each well of a 96-well cell culture plate and allowed to adhere for 12 h before drug addition, while suspended cells were seeded just before drug addition, both with an initial density of 1 × $10^5$ cells/mL in 100 $\mu$L of medium. Each tumor cell line was exposed to the test compound at various concentrations in triplicate for 48 h, with DDP and toxal as positive controls. After the incubation, MTT (100 $\mu$g) was added to each well, and the incubation continued for 4 h at 37 °C. The cells were lysed with 200 $\mu$L SDS after the removal of 100 $\mu$L of medium. The optical density of the lysate was measured at 595 nm in a 96-well microtiter plate reader (Bio-Rad 680). The $IC_{50}$ value was calculated by the Reed and Muench's method.[10]

## Electronic Supplementary Material

Supplementary material is available in the online version of this article at http://dx.doi.org/10.1007/s13659-013-0024-9 and is accessible for authorized users.

## Acknowledgments

The authors are grateful to Dr. Xiao-Hua Jin of Institute of Botany, Chinese Academy of Sciences, for identification of the plant. This work was financially supported by the National Natural and Science Foundations of China (No. 30800090), "Xi-Bu-Zhi-Guang" project (No. 2009312D11017) from Chinese Academy of Sciences and the Fund of the State Key Laboratory of Phytochemistry and Plant Resources in West China (P2010-ZZ012).

**Open Access** This article is distributed under the terms of the Creative Commons Attribution License which permits any use, distribution, and reproduction in any medium, provided the original author(s) and source are credited.

## Electronic supplementary material

Supplementary material, approximately 1.63 MB.

Supplementary material, approximately 16.6 KB.
