# Supplementary material for: Dendrowardol C, a novel sesquiterpenoid from Dendrobium wardianum Warner
Source: Nat Prod Bioprospect. 2013 Apr 24;3(3):89–92. doi: 10.1007/s13659-013-0024-9 (PMC4131667; doi:10.1007/s13659-013-0024-9)
Supplement: Supplementary file 1 — Supplementary material, approximately 1.63 MB. [file 13659_2013_24_MOESM1_ESM.pdf]

Electronic Supplementary Material

Dendrowardol C, a novel sesquiterpenoid from *Dendrobium wardianum* Warner

Wei-Wei FAN,[a,b] Feng-Qing XU,[a,b] Fa-Wu DONG,[a,b] Xiao-Nian LI,[a] Yan LI,[a] Yu-Qing LIU,[a] Jun ZHOU,[a] and Jiang-Miao HU,[a,]*

[a]State Key Laboratory of Phytochemistry and Plant Resources in West China, Kunming Institute of Botany, Chinese Academy of Sciences, Kunming 650201, China

[b]University of Chinese Academy of Sciences, Beijing 100049, China

Received 17 March 2013; Accepted 2 April 2013

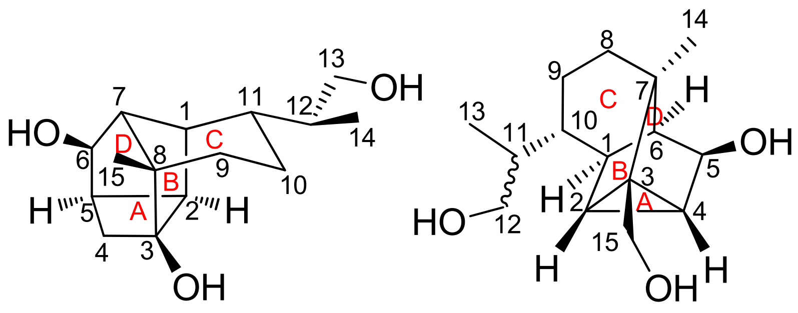

1 **2**

Structures of compounds **1** and **2**

*To whom correspondence should be addressed. E-mail: hujiangmiao@mail.kib.ac.cn

Electronic Supplementary Material

Figure S1. ^{1}H NMR spectrum of dendrowardol C (**1**) in pyridine-d_5

Figure S2. ^{13}C NMR spectrum of dendrowardol C (**1**) in pyridine-d_5.

Figure S3. HSQC spectrum of dendrowardol C (**1**) in pyridine-d_5.

Figure S4. HMBC spectrum of dendrowardol C (**1**) in pyridine-d_5.

Figure S5. ^{1}H–^{1}H COSY spectrum of dendrowardol C (**1**) in pyridine-d_5.

Figure S6. ROESY spectrum of dendrowardol C (**1**) in pyridine-d_5.

Figure S7. EIMS spectrum of dendrowardol C (**1**).

Figure S8. HRESI(+)MS spectrum of dendrowardol C (**1**).

Figure S9. IR spectrum of dendrowardol C (**1**).

Figure S10. UV spectrum of dendrowardol C (**1**).

Figure S11. ^{1}H NMR spectrum of compound **2** in pyridine-d_5.

Figure S12.^{13}C NMR spectrum of compound **2** in pyridine-d_5.

Figure. S13. UPLC-MS spectrum of detecting **1** in the original ethanol extract.

S1. X-ray crystal data for Dendrowardol C (**1**).

Figure S1. ^{1}H NMR spectrum of dendrowardol C (**1**) in pyridine-d_5.

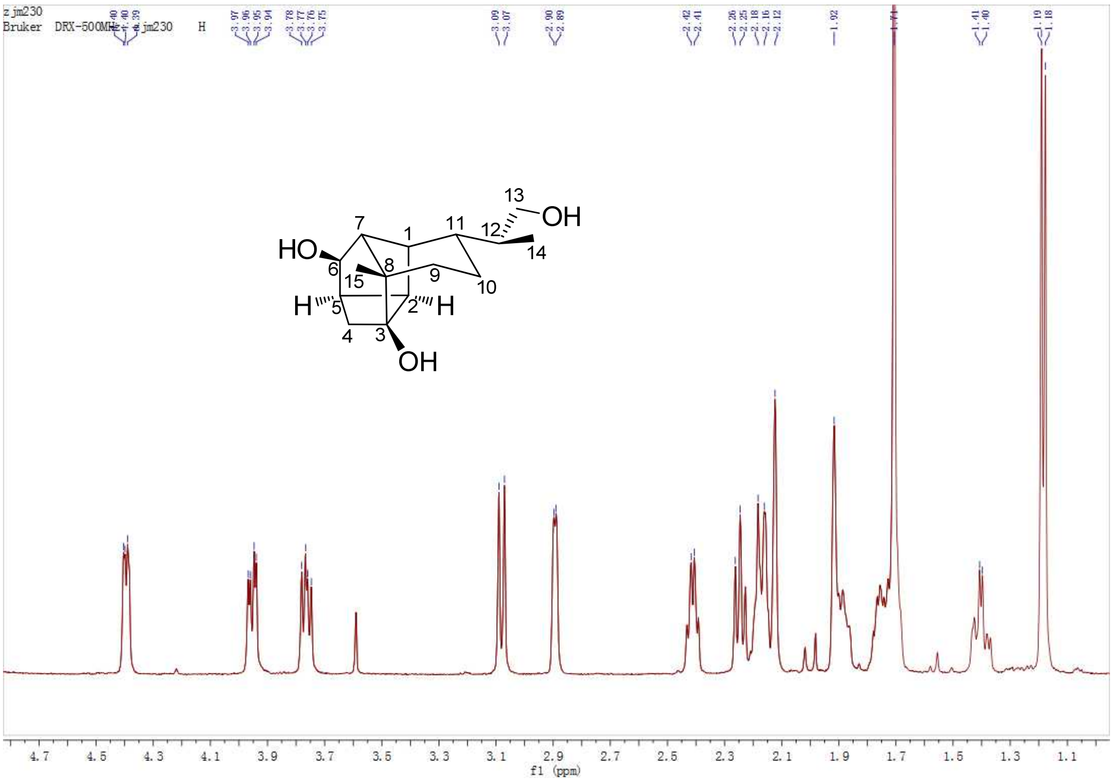

Figure S2. ^{13}C NMR spectrum of dendrowardol C (**1**) in pyridine-d_5.

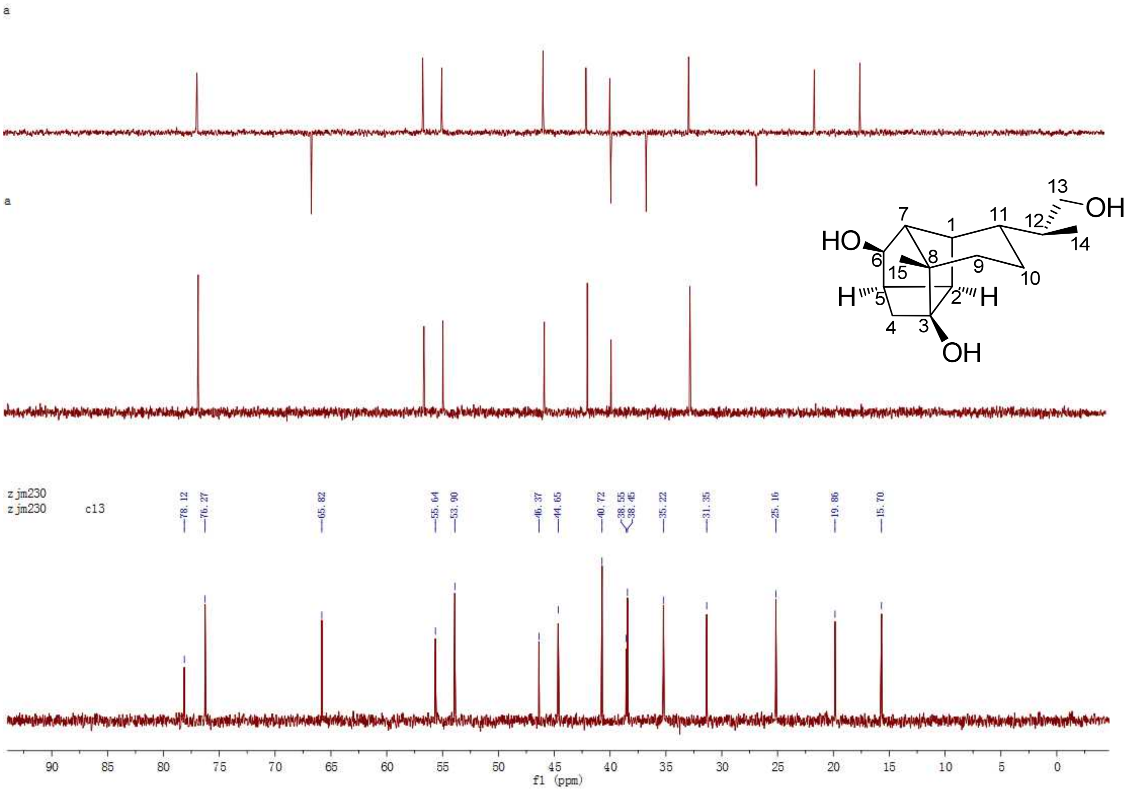

Figure S3. HSQC spectrum of dendrowardol C (**1**) in pyridine-d_5.

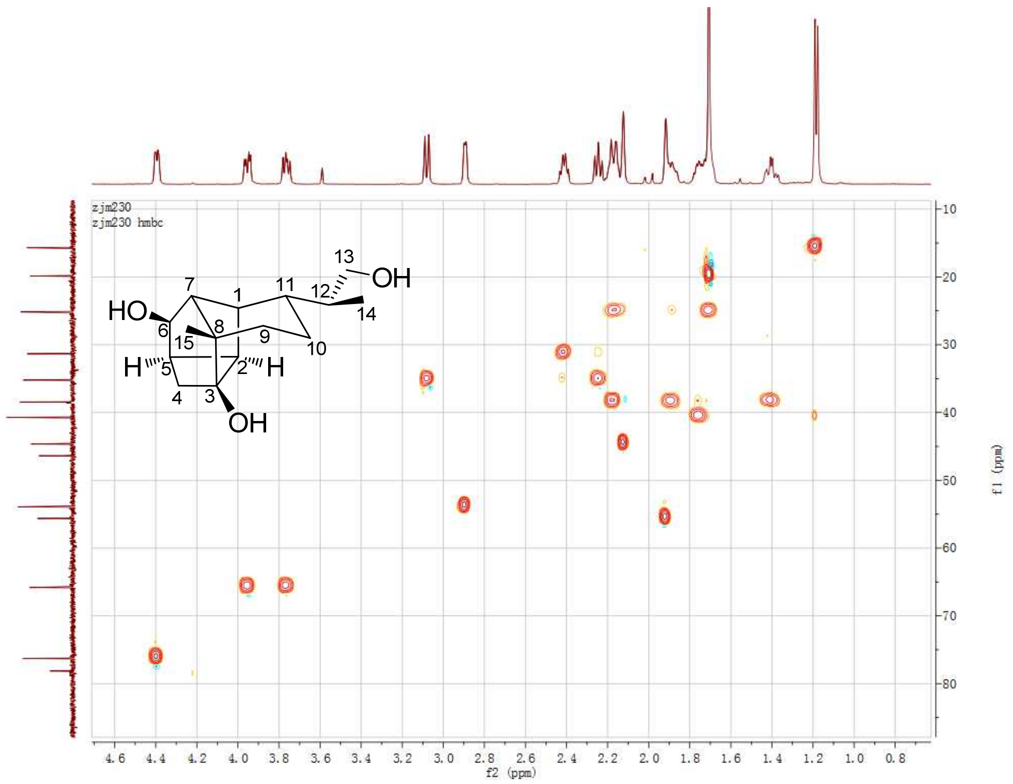

Figure S4. HMBC spectrum of dendrowardol C (**1**) in pyridine-d_5.

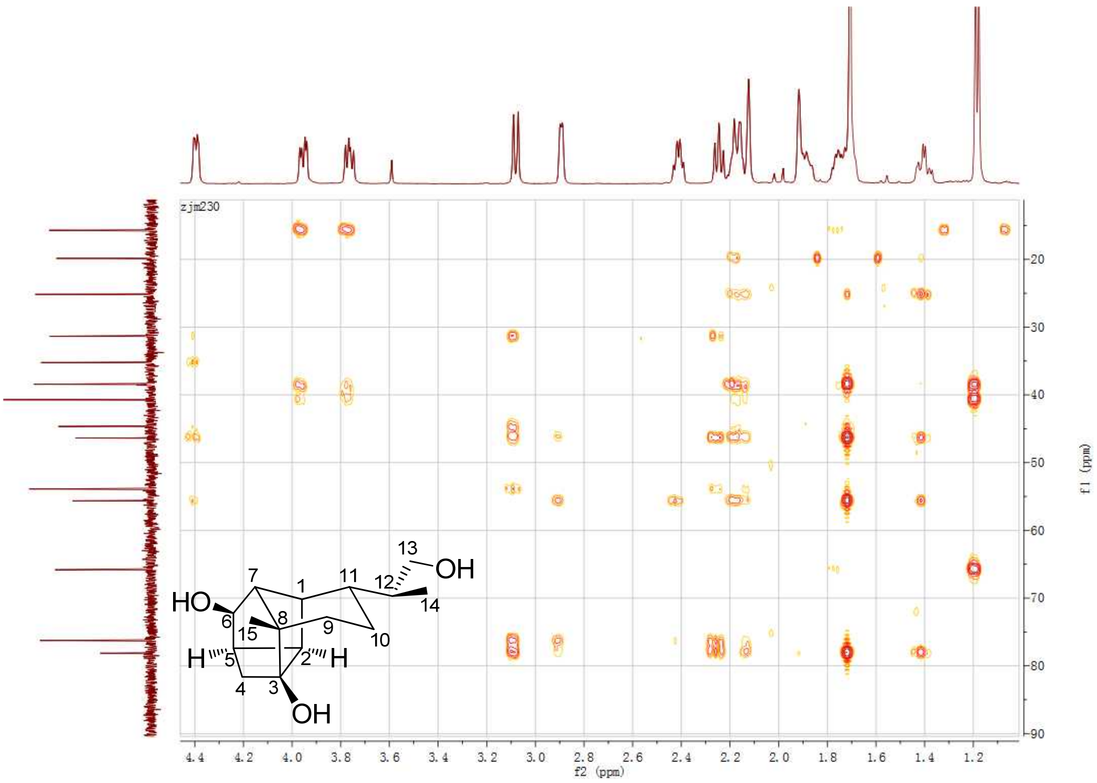

Figure S5. ^{1}H–^{1}H COSY spectrum of dendrowardol C (**1**) in pyridine-d_5.

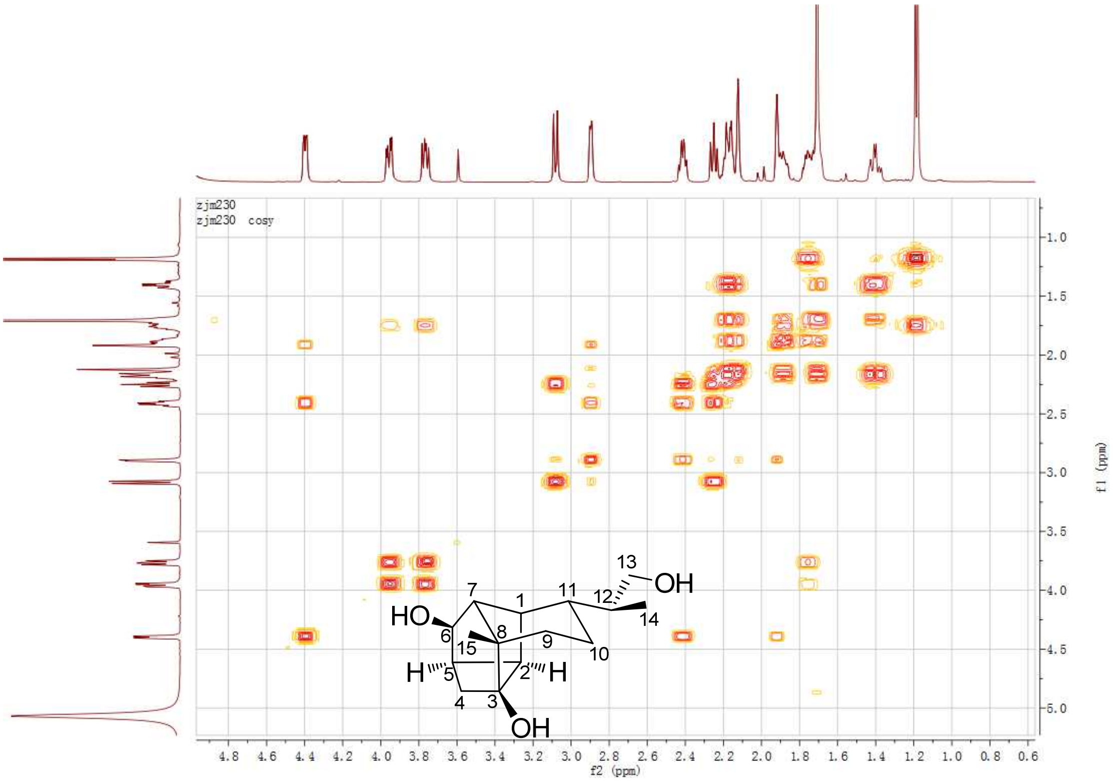

Figure S6. ROESY spectrum of dendrowardol C (**1**) in pyridine-d_5.

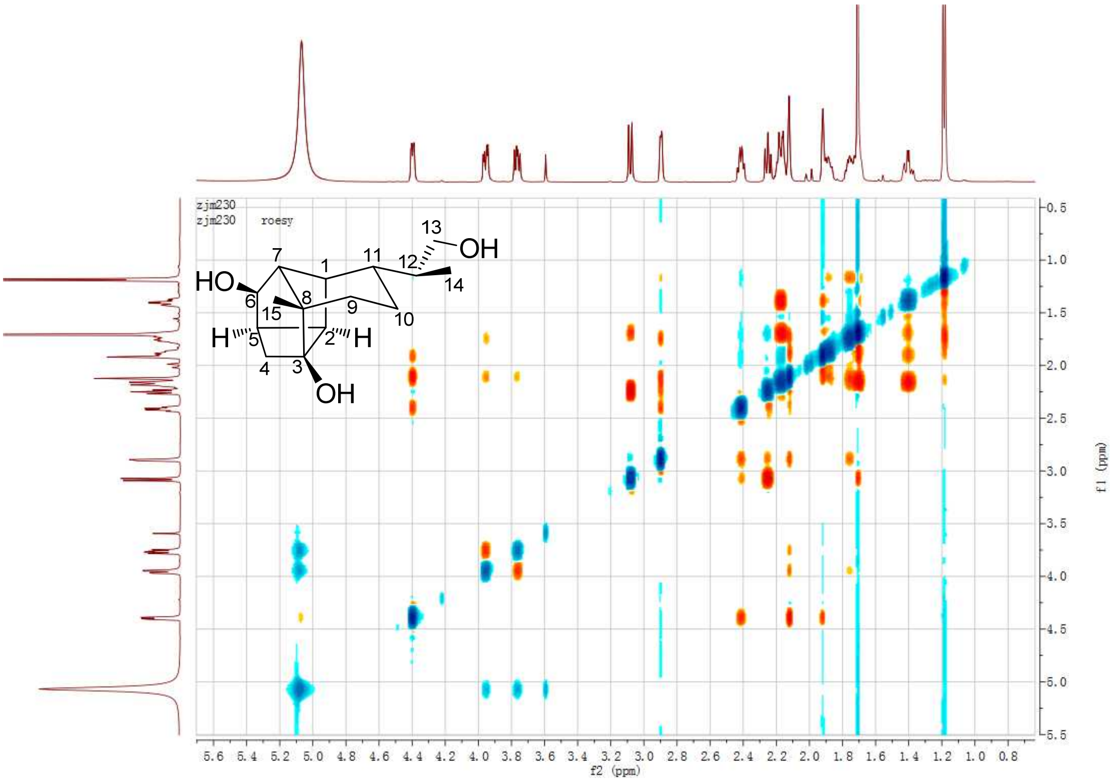

Figure S7. EIMS spectrum of dendrowardol C (**1**).

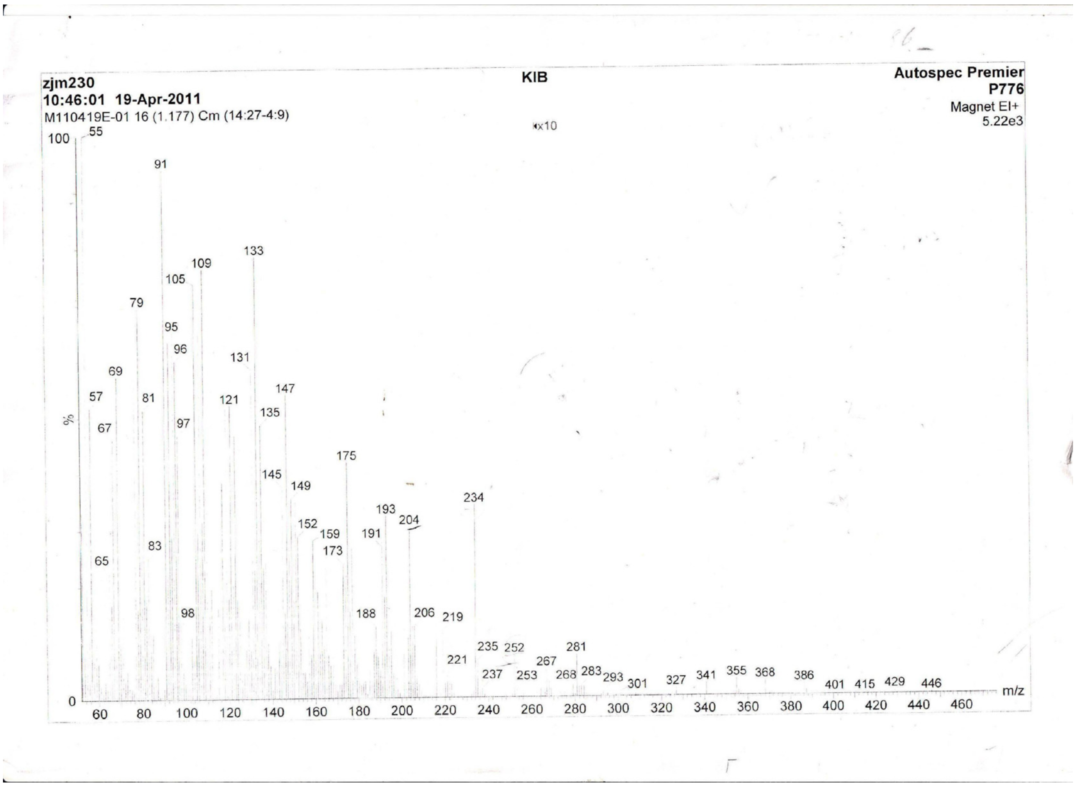

Figure S8. HR-ESI(+)MS spectrum of dendrowardol C (**1**).

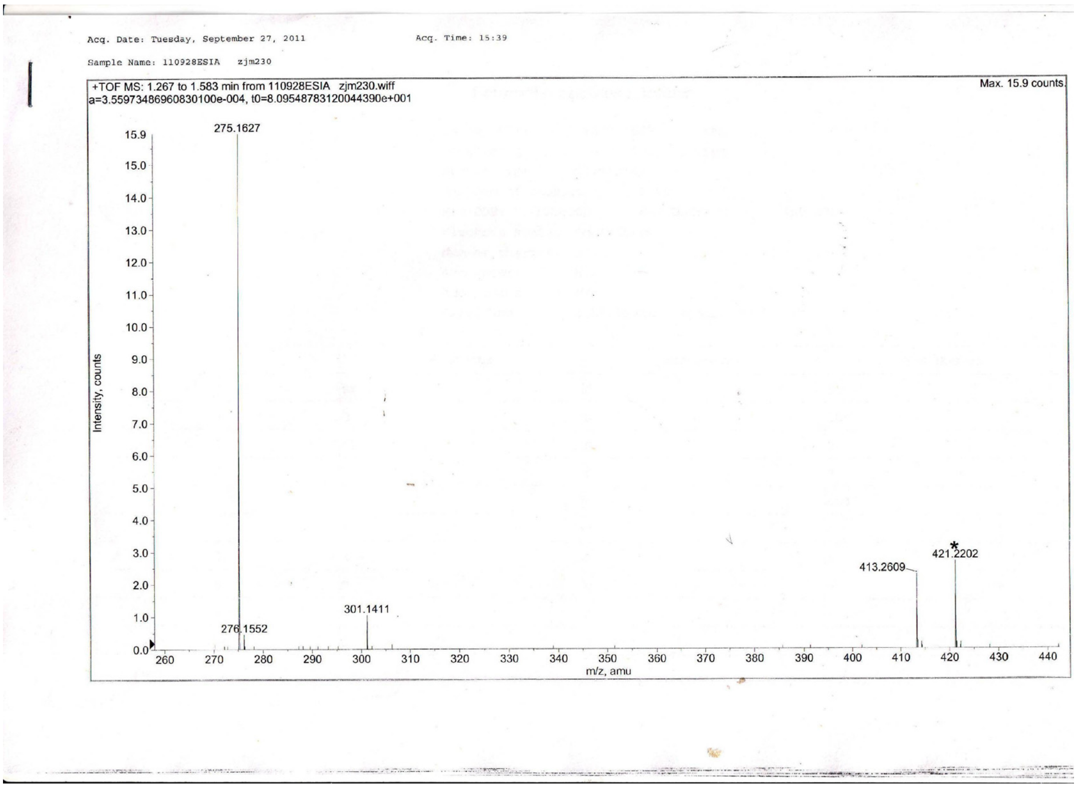

Figure S9. IR spectrum of dendrowardol C (**1**).

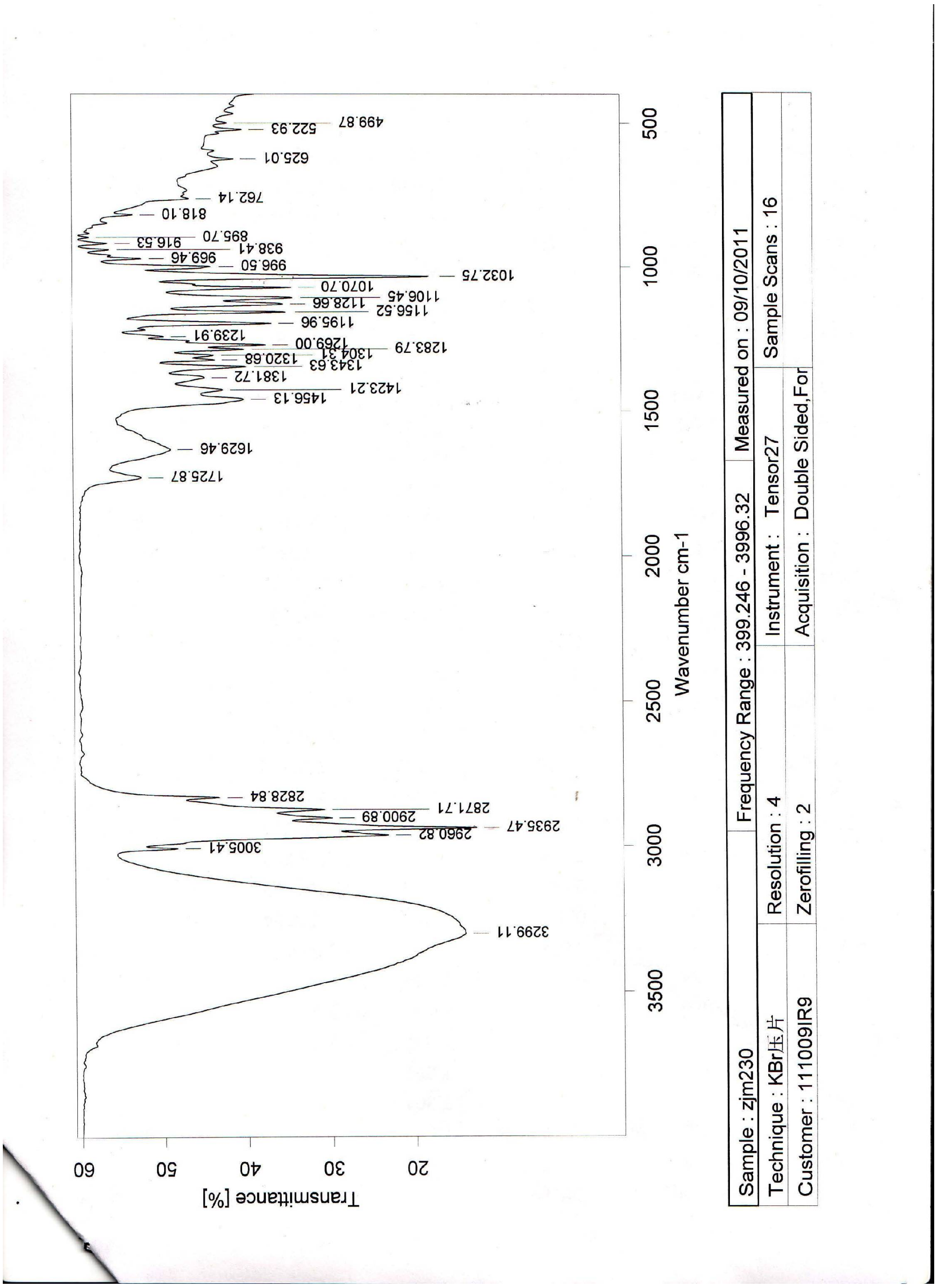

Figure S10. UV spectrum of dendrowardol C (**1**).

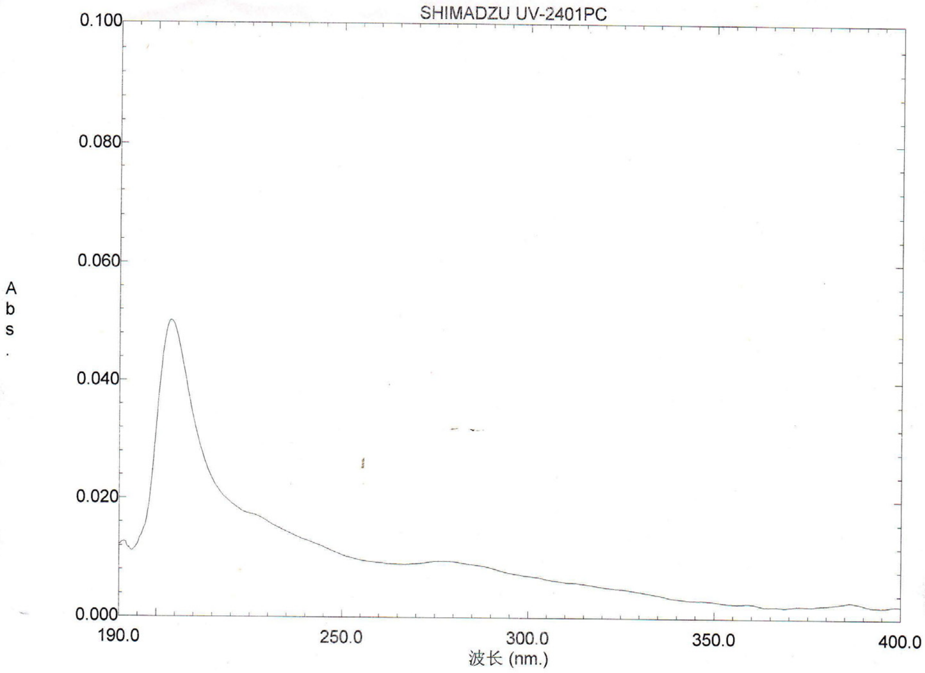

文件名: ZJM230

ZJM230 ———

创建于: 19:08 11-07-22
数据: 原始

样品浓度：0.0319毫克/毫升
溶剂：甲醇

测量模式: Abs.
扫描速度: 中速
狭缝: 5.0
采样间隔: 0.2

否.	波长 (nm.)	Abs.
1	276.00	0.0095
2	203.80	0.0502

Figure S11. ^{1}H NMR spectrum of compound **2** in pyridine-d_5.

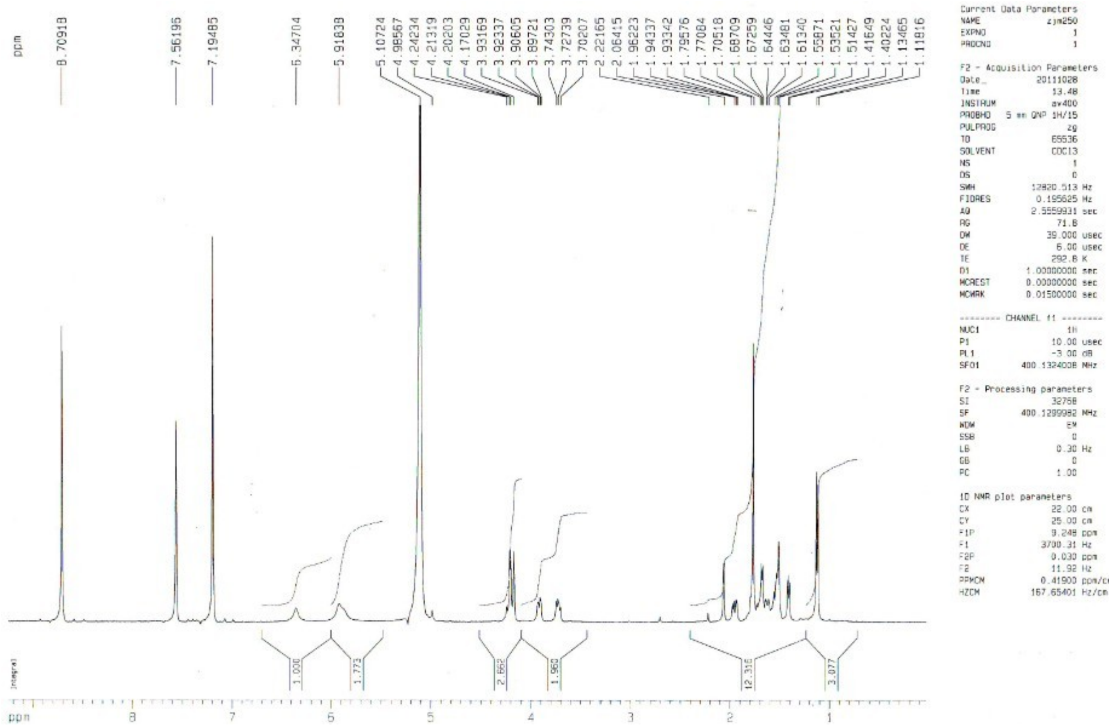

Figure S12. ^{13}C NMR spectrum of compound **2** in pyridine-d_5.

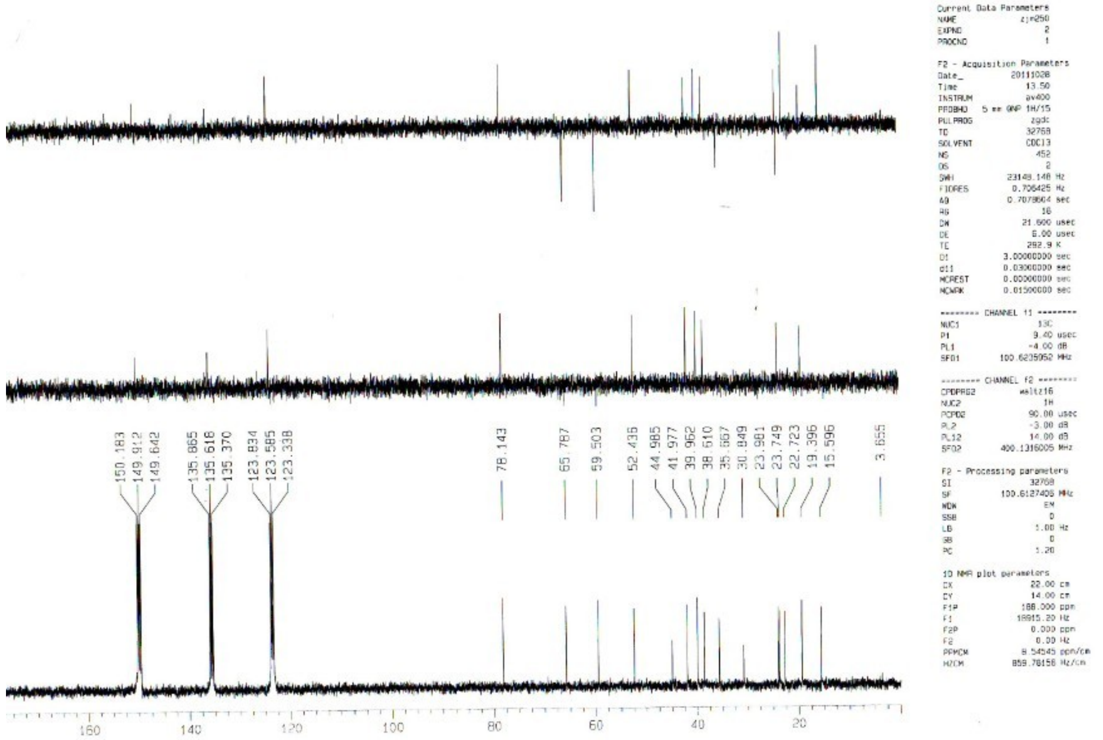

S1. X-ray crystal data for dendrowardol C (**1**).

Identification code	1 _Cu
Empirical formula	C15 H24 O3
Formula weight	252.34
Temperature	100(2) K
Wavelength	1.54178 A
Crystal system, space group	Orthorhombic, P 21 21 21
Unit cell dimensions	a = 6.39120(10) A alpha = 90 deg.
	b = 11.8518(2) A beta = 90 deg.
	c = 18.2414(3) A gamma = 90 deg.
Volume	1381.74(4) A^3
Z, Calculated density	4, 1.213 Mg/m^3
Absorption coefficient	0.659 mm^-1
F(000)	552
Crystal size	0.90 x 0.19 x 0.18 mm
Theta range for data collection	4.45 to 69.59 deg.
Limiting indices	-7<=h<=7, -14<=k<=13, -22<=l<=21
Reflections collected / unique	13935 / 2522 [R(int) = 0.0384]
Completeness to theta = 69.59	98.3 %
Absorption correction	Semi-empirical from equivalents
Refinement method	Full-matrix least-squares on F^2
Data / restraints / parameters	2522 / 0 / 169
Goodness-of-fit on F^2	1.050
Final R indices [I>2sigma(I)]	R1 = 0.0310, wR2 = 0.0814
R indices (all data)	R1 = 0.0311, wR2 = 0.0815
Absolute structure parameter	0.00(18)
Extinction coefficient	0.0085(7)
Largest diff. peak and hole	0.213 and -0.194 e.A^-3
